# Differential effects of exposure to cooperative versus competitive games on sharing behavior in young children

**DOI:** 10.3389/fpsyt.2025.1545932

**Published:** 2025-07-03

**Authors:** Qian Zhang, JiaLe Ruan, DingYong Xiong

**Affiliations:** ^1^ Center for Studies of Education and Psychology of Ethnic Minorities in Southwest Area & Division of Early Childhood Education at Faculty of Education, Southwest University, Chongqing, China; ^2^ Department of Preschool Education, South Sichuan Preschool Education College, Sichuan, China; ^3^ College of Education, Sichuan Institute of Industrial Technology, Sichuan, China

**Keywords:** competitive games, cooperative games, sharing behavior, young children, game settings

## Abstract

**Background:**

Previous research has documented a relationship between prosocial video games and prosocial behaviors. However, there has been much less evidence on the potential effects of real-life prosocial games. Theoretically, games in which characters cooperate and help each other in nonviolent ways should increase prosocial behavior.

**Objective:**

In this study, we experimentally evaluated the effects of competitive and cooperative games on sharing behavior.

**Methods:**

The study sample were 120 children (*M*_age_ = 4.73 years, *SD* = 0.49) from two kindergartens in China. Participants were randomly assigned to play the same game for 15 minutes in either a cooperative or competitive setting. Then their sharing behaviors were measured. A 2(Setting) × 2 (Gender) Analysis of Covariance (ANCOVA) was performed, controlling for age.

**Results:**

Results indicated that playing a cooperative game (versus competitive game) increased sharing behavior. In addition, we observed a Setting × Gender interaction. Basically, boys’ sharing attitudes and behaviors were unaffected by the Setting manipulation, whereas girls’ were affected. Similarly, the competitive setting increased girls’ sharing attitudes and behaviors, whereas the boys’ was relatively unaffected. Precisely, girls reported more sharing behavior than boys in competitive gaming condition.

**Conclusion:**

Findings of this study suggest that educational practitioners can utilize a cooperative game (versus competitive game) as an effective way to develop sharing behavior among kindergarten children. Boys should be a target group for sharing behavior development, especially in a competitive game setting.

## Introduction: Effects of cooperative versus competitive games on sharing behavior in young children

Previous research has shown that playing violent video games cooperatively in a team increases subsequent cooperative behavior (1, 2), while playing competitive video games increases aggression (3, 4). However, existing studies have not clarified the specific effects of competitive and cooperative settings in the same game on children’s sharing behavior. Sharing is typically defined as sacrificing a child’s ownership to benefit others, such as giving away a toy they are playing with to another child (5). Shared materials such as food, stickers and toys can become valuable items for children (6–10). Since sharing attitude is an internal mental state that decides whether to allocate resources with others (11, 12). Since sharing attitude is an internal mental state that decides whether to allocate resources with others (11, 12), we assessed sharing attitudes by asking young children if they were willing to allocate their stickers to other peers who also liked them. Based on this, children’s sharing attitude refers to their willingness to allocate stickers to others; children’s sharing behavior refers to the number of stickers actually allocated by children to others. Although children around the world play games, there has been relatively little experimental research on the effects of competitive versus cooperative games on children’s sharing behavior. More importantly, we attempt to advance this field by using the same game in two different settings (competitive versus cooperative) to achieve better ecological validity than the previous research. Thus, this is the main reason why we conducted this experimental study, and the topic of this work is also very important and necessary.

### Social interdependence theory

Social Interdependence Theory posits that social interdependence exists when people have common goals, and that each person’s behavior is influenced by the behavior of others. Different types of interdependence can produce various behavioral outcomes (13, 14). Specifically, playing a prosocial game (positive interdependence) increases prosocial behavior in college students (15), and playing prosocial video games increases prosocial behavior 3 to 4 months later in 5th graders (16). In contrast, negative interdependence (e.g., competition) increases children’s aggressive behavior (17). Previous researchers have found that setting the cooperative mode of the Wii Sports Resort canoeing allows the team to work together to accomplish navigation goals, thus triggering spontaneous helping behaviors (18). However, long-term exposure to competitive video games and competitive gambling can lead to aggression (19, 20). In view of this, we attempt to divide a game into either a competitive or a cooperative setting, and investigate how these two separate game settings affect children’s sharing behavior. Although Social Interdependence Theory was mainly applied to groups, we attempt to explain the effects of subsidies and cooperation on sharing behavior among young children and find out how this theory applies to 4–5 year olds in China.

### Competitive game, cooperative game, and prosocial behavior

Previous findings suggest that children in a competitive context are likely to show a stronger preference for discarding resources to avoid inequity (21). However, studies have also shown that children’s generosity is more likely to decline when resources are allocated in a competitive rather than cooperative setting (11, 22, 23). In addition, the amount of time spent playing video games in prosocial settings is positively correlated with prosocial behaviors such as sharing and helping (16). Similarly, cooperative gaming increase subsequent spontaneous helping behavior and cooperative behavior (2, 18, 24, 25). Thus, exposure to cooperative or competitive games may influence children’s sharing behavior.

### Gender and sharing behavior

There are gender differences in altruistic behavior (26–28). More specifically, girls engage in more sharing behaviors than boys (e.g., 29–32). Conversely, other scholars have concluded that boys are more generous in sharing behavior than girls because Chinese culture emphasizes the traditional notion that boys are more generous than girls (33, 34). However, our experimental results are inconsistent with the previous literature above, we have included this contradiction in the revision. In addition, researchers claim that gender per se has no significant effect on sharing behavior in economic games (11, 35, 36). In light of this, there is controversy about the gender effects on children’s sharing behavior, which is worth further exploration. Therefore, it is necessary to test the gender effects on Chinese children via experiments for an exploratory purpose.

### Age and sharing behavior

A growing body of researches suggests that children become more generous as they get older, and that younger children are more likely to exhibit selfish behavior than older children (e.g., 22, 30, 37, 38). However, other researchers have not found significant differences in sharing behavior by age (11, 39). However, other researchers have not found significant differences in sharing behavior by age (11, 39). It is worth noting that the age range of the children in this study is restricted, so we drop the age effects on children’s sharing behavior.

### The present study

Taken together, the primary objective of this study is to examine the causal relationship between competitive and cooperative gameplay and sharing behavior. The main research questions of this study are as follows: (a) How does exposure to the same game either in a cooperative or competitive setting affect children’s sharing behavior? (b) If so, are there gender effects on children’s sharing behavior, immediately after exposure to games? (c) Is there a significant association between sharing attitude and sharing behavior? In response to these research questions, we suggest the following hypotheses:

H1: *Exposure to a cooperative game will increase children’s sharing behavior*.
H2: *Boys will show higher levels of sharing attitude and sharing behavior than girls in a cooperative game setting*.
H3: *Sharing attitude will be significantly related to sharing behavior*.

## Method

### Participants

The study was taken place in Spring semester of 2019. We recruited 120 children aged between 4 and 5 years (*M*_age_ = 4.73, *SD* = 0.49; 50% girls) from two kindergartens in Southwest of China. 4 years olds were born between September 2015 and September 2016, and 5 years olds were born between September 2014 and September 2015. Participants were randomly assigned to play the game in a cooperative context or competitive setting. According to our interviews, none of them have once played this game. Parents gave informed consents to their children’s participation, and the consent rates reached 100%. No participants failed to complete the experiment.

### Design

We conducted a 2 (Setting: competitive vs. cooperative) x 2 (Gender: boys vs. girls) between-subjects design. The independent variables are setting and gender. The dependent variables are sharing attitude (willingness to allocate stickers with others) and sharing behavior (the number of stickers the children actually gave to others).

### Materials

*Games.* To conduct a manipulation check, we initially invited experts to rate three games: *Pony Crossing*, *Pinball* and *Fishing*, in order to choose the most appropriate one. We chose them because they meet the requirements of both competition and cooperation. *Pony Crossing* requires children to cross a distance of about 15 meters in 15 minutes. This game is developed on the basis of cushions commonly used in kindergarten activities. Half of the participants played the same game in a competitive or cooperative setting. In a competitive setting, a pair of children use two cushions to form a route. Participants need to step on this route and pass 15 meters. Whoever passes first wins. In a cooperative setting, a pair of children use three cushions together to pass a relay (i.e., one person makes the road, one person steps on the cushion), forming a 15-meter route. If they successfully pass within 15 minutes, they win. Otherwise, they will lose the game. *Pinball* requires children to score points by manipulating marbles into holes of the appropriate color after playing five rounds in 15 minutes. The two participants who first enter the colored hole by manipulating a marble earn 1 point, while the participant who accumulates the most points over the five rounds wins. After that, a pair of participants cooperate to control the entry of a marble into a hole of the corresponding color, and there is either a winner or a loser in the end. The pair of winners are the contestants who score more than 3 points in 5 rounds. *Fishing* requires children to fish with a rod in 15 minutes. we used 10 plastic fish as materials in a circle with a fence. The children fished with the simple fishing rods. The game is over as soon as the fish is caught. Children who successfully catch more fish within 15 minutes are winners in a competitive setting, whilst children who cooperate with each other to catch all the fish in 15 minutes are winners in a cooperative setting. Notably, participants have never played the three games. Each of the three games was limited to 15 minutes. We set up a unified practice session and help children understand the rules of the games.

To control for potential confounding variables, we invited 40 postgraduates in child psychology (50% girls) to rate these games in terms of Competition, Cooperation, Interest, Pleasure, and Difficulty (cf. 40). The experimental assistant orally explained the rating dimensions by using a five-point Likert scale (*1 = not consistent at all, 5 = very consistent*). One-way analysis of variance (ANOVA) was performed to test the differences in ratings across three games based on five dimensions (**Table 1**). In other words, the ratings include both the competitive or the cooperative versions of the three games. *Pony Crossing* scored higher than the other two games in Competition [*F*(2,117) = 9.53, *p* < 0.001, *d* = 0.60; partial η^2^ = 0.08; *M _Pony Crossing_* = 3.88 (*SD_Pony Crossing_* = 0.79) > *M _Pinball_* = 3.35 (*SD _Pinball_* = 0.66) > *M _Fishing_* = 3.23 (*SD _Fishing_* = 0.66)] and Cooperation [*F*(2,117) = 7.84, *p* = 0.001, *d* = 0.55; partial η^2^ = 0.07; *M_Pony Crossing_* = 3.95 (*SD_Pony Crossing_* = 0.85) > *M _Pinball_* = 3.55 (*SD _Pinball_* = 0.75) > *M _Fishing_* = 3.30 (*SD _Fishing_* = 0.61)]. However, no significant differences in Interest [*F*(2,117) = 1.38, *p* = 0.26, *d* = 0.23; partial η^2^ = 0.01], Pleasure [*F*(2,117) = 1.13, *p* = 0.33, *d* = 0.20; partial η^2^ = 0.01] and Difficulty [*F*(2,117) = 1.99, *p* = 0.14, *d* = 0.28; partial η^2^ = 0.02] were found. As a result, *Pony Crossing* was chosen as the formal game material for the follow-up experiment.

**Table 1:** Rating results of game materials.

Dimension	Pony Crossing *(M ± SD)*	Pinball*(M ± SD)*	Fishing*(M ± SD)*	*F*	*d*
Interest	4.08 ± 0.66	3.83 ± 0.68	3.98 ± 0.70	1.38	0.23
Pleasure	4.12 ± 0.69	3.90 ± 0.63	3.95 ± 0.78	1.13	0.20
Difficulty	2.93 ± 0.63	3.23 ± 0.66	3.10 ± 0.74	1.99	0.28
Competition	3.87 ± 0.79	3.35 ± 0.66	3.23 ± 0.66	9.53***	0.60
Cooperation	3.95 ± 0.85	3.55 ± 0.75	3.30 ± 0.61	7.84**	0.55

**p < 0.01, ***p < 0.001.

*Sharing Materials.* Candy, stickers, storybooks, crayons and Gashapon toys were chosen as sharing materials, which were similar to the toys they used in real life and reflected the real needs of the children. At the same time, we invited 10 children aged 4–5 to rate the shared material to avoid some potential confounding factors (i.e., item preferences). Then we chose the most appropriate sharing material to measure children’s sharing behavior. Children assessed for sharing materials will be excluded from the experiment to avoid the confounding effects of the experiment. The lab assistant paired the five shared materials with color pictures and asked the children to rate them on a 5-point Likert scale (1 = very dislike, 5 = very like). The higher the score, the most popular material to share. One-way ANOVA was used to compare differences in affection of the five shared items. Children liked stickers most [*F*(4,45) = 4.85, *p* = 0.002, *d* = 0.80; *M _stickers_* = 4.30 (*SD _stickers_* = 0.67) > *M _Gashapon toys_* = 3.80 (*SD _Gashapon toys_* = 1.23) > *M _Candy_* = 3.50 (*SD _Candy_* = 0.71) > *M _crayons_* = 3.20 (*SD _crayons_* = 0.92) > *M _storybooks_* = 2.70 (*SD _storybooks_* = 0.67)]. Therefore, we chose stickers as the most appropriate material to share.

### Sharing attitude

To measure sharing attitudes, we took each participant to a separate room immediately after playing a given game in either a competitive or cooperative setting (i.e. two participants who were independent of each other and did not interfere with each other) and rewarded them with five stickers after the game to thank them for their cooperation. Participants did not know the other children’s actual reward. In addition, we created a situation where another child in the game also liked stickers and we gave participants the choice of whether and how much to share. We then measured children’s sharing attitudes by asking the following questions: “Are you willing to allocate your stickers to another child who plays with you? Because he or she really likes your stickers.” Throughout the process, the lab assistant asked each child uniform questions (i.e., the questions above) without leading the language. We rate the sharing attitude as zero if the child is unwilling to share, and we rate sharing attitude as 1 point if the child is willing to allocate.

### Sharing behavior

Sharing behavior refers to the number of stickers that children actually share with others. The stickers here are the same as in the sharing attitude measurement. The lab assistant could observe the children assigning stickers to other people participating in the game (e.g., one sticker gets 1 point, no stickers get 0 points, and five stickers get 5 points). More stickers children voluntarily allocated to others were indicative of more sharing behaviors, and vice versa. Of course, no sharing behavior was available if the number of stickers was zero. In this study, we gave each child 5 stickers to allow him/her to spontaneously decide how many stickers they would like to give others. In fact, children did not know whether other children had rewards to measure their real sharing behavior (i.e., not affected by the number of stickers from the other side). The more stickers the children voluntarily allocated to others, the more they shared, and vice versa. Of course, if the sticker count is zero, there is no sharing behavior. In this study, we gave each child five stickers and asked him or her to spontaneously decide how many stickers to give to others. In fact, the children didn’t know whether the others had a reward to measure their true sharing behavior (not affected by the number of stickers).

### Procedure

First, parents gave informed consent to their children’s voluntary participation before the study began. Second, 60 children either played the game in either a designated competitive or cooperative setting. Based on previous research (41), the game *Pony Crossing* was played for 15 minutes in each setting. The lab assistant sat on hand to answer questions raised by children and make sure they completed the assigned game. Third, sharing attitudes were measured by self-report and sharing behavior was measured by the number of stickers the children allocated to others. Finally, we thanked each child for their cooperation by giving him/her nice gifts (e.g., Ultraman, Barbie dolls).

### Data analysis

A 2 (Setting) x 2 (Gender) Analysis of Covariance (ANCOVA) were performed by SPSS 21.0, with age (continuous variable) included as a covariate. Specifically, we aimed to investigate the main effect of game setting and gender on sharing attitudes and behaviors, and how they interact with each other.

## Results

### Descriptive statistics

**Tables 2**, **3** listed the means, standard deviations and cell sample sizes of sharing attitudes and sharing behaviors in cooperative and competitive game settings. Overall, there was more sharing attitude and sharing behavior in the cooperative setting than in the competitive setting. As to gender, girls showed more sharing attitude and sharing behavior than boys in the competitive setting. Thus, game setting and gender may be related to sharing attitudes and sharing behaviors, which suggests that we should specifically analyze the effect of the main variables.

**Table 2:** Descriptive statistics for children’ sharing attitude (N = 120).

Gender	Competitive (*M ± SD*)	*N*	Cooperative (*M ± SD*)	*N*
Boys	0.47 ± 0.51	30	0.83 ± 0.38	30
Girls	0.83 ± 0.38	30	0.80 + 0.41	30
Total	0.65 ± 0.48	60	0.82 + 0.39	60

**Table 3:** Descriptive statistics for children’ sharing behavior (N = 120).

Gender	Competitive (*M ± SD)*	*N*	Cooperative (*M ± SD)*	*N*
Boys	1.10 ± 0.84	30	2.67 ± 1.42	30
Girls	2.67 ± 1.52	30	2.17 ± 1.02	30
Total	1.88 ± 1.45	60	2.42 ± 1.25	60

### Analysis of covariance on sharing attitude

**Figure 1:**
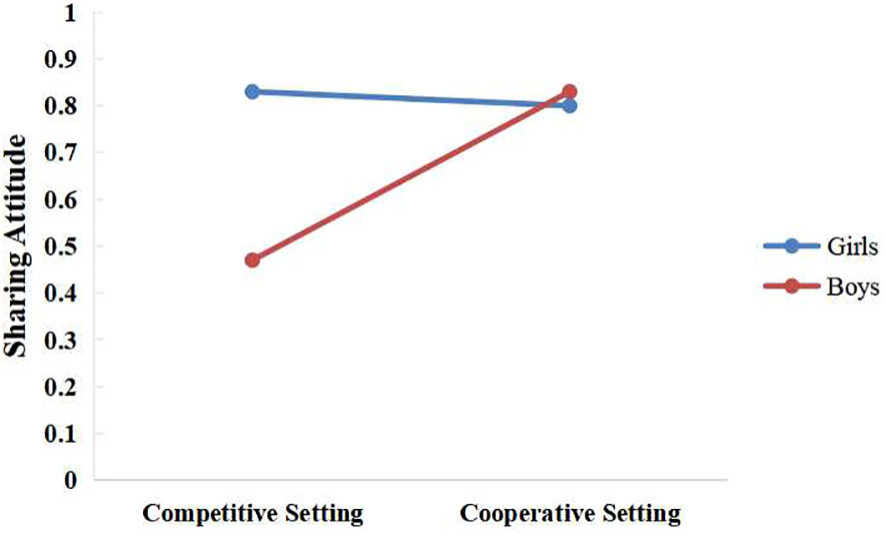
Interaction between setting and gender on sharing attitude.

The main effect of setting on sharing attitude was significant [*F*(1,115) = 4.65, *p* = 0.03, *d* = 0.40, partial η^2^ = 0.04]. Playing a game in a cooperative setting led to higher sharing attitude than playing a game in a competitive setting [*M _cooperative_* = 0.82 (*SE _cooperative_* = 0.06) > *M _competitive_* = 0.65 (*SE _competitive_* = 0.06)]. The main effect of gender on sharing attitude was significant [*F*(1,115) = 4.65, *p* = 0.03, *d* = 0.40, partial η^2^ = 0.04]. Girls showed higher sharing attitude than boys [*M _girls_* = 0.82 (*SE_girls_* = 0.06) > *M _boys_* = 0.65 (*SE_boys_* = 0.06)]. In addition, the setting x gender interaction on sharing attitude was significant [*F*(1,115) = 6.70, *p* = 0.01, partial η^2^ = 0.05]. A simple effect analysis indicated that girls displayed higher sharing attitude than boys in the competitive game setting [*F*(1,115) = 11.25, *p* = 0.001, partial η^2^ = 0.09; *M* = 0.83 (*SE* = 0.08) > *M* = 0.47 (*SE* = 0.08)], whereas no significant gender effects on sharing attitude were found in the cooperative game setting [*F*(1,115) = 0.09, *p* = 0.76, partial η^2^ < 0.001] (**Figure 1**).

### ANCOVA on sharing behavior

**Figure 2:**
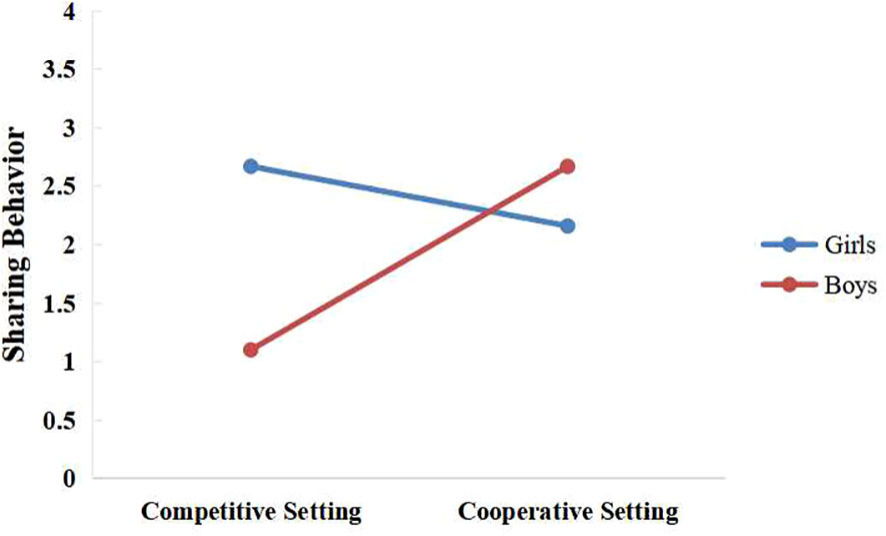
Interaction between setting and gender on sharing behavior.

The main effect of setting on sharing behavior was significant [*F*(1,115) = 5.72, *p* = 0.02, *d* = 0.44, partial η^2^ = 0.05]. Playing a game in a cooperative setting led to more sharing behavior than playing a game in a competitive setting [*M _cooperative_* = 2.42 (*SE _cooperative_* = 0.16) > *M _competitive_* = 1.88 (*SE _competitive_* = 0.16)]. The main effect of gender on sharing behavior was significant [*F*(1,115) = 5.70, *p* = 0.02, *d* = 0.44, partial η^2^ = 0.05]. Girls displayed more sharing behavior than boys [*M _girls_* = 2.42 (*SE _girls_* = 0.16) > *M _boys_* =1.88 (*SE _boys_* = 0.16)]. In addition, the setting x gender interaction on sharing behavior was significant [*F*(1,115) = 21.88, *p* < 0.001, partial η^2^ = 0.16]. A simple effect analysis indicated that girls exhibited more sharing behavior than boys in the competitive game setting [*F*(1,115) = 24.95, *p* < 0.001, partial η^2^ = 0.17; *M* = 2.67 (*SE* = 0.22) > *M* = 1.10 (*SE* = 0.22)], whereas no significant gender effects on sharing behavior were found in the cooperative game setting [*F*(1,115) = 2.63, *p* = 0.11, partial η^2^ = 0.02] (**Figure 2**).

### The correlation between sharing attitude and sharing behavior

Sharing attitude was positively correlated with sharing behavior [*r* = 0.22, *p* = 0.02].

## Discussion

Our results support the first hypothesis, which suggests that children in a cooperative setting report more higher levels of attitudes and behaviors than children in a competitive setting. This finding also replicates previous research that suggests sharing behavior is based more on cooperation than competition in games (23, 42, 43). Meanwhile, our results show that positive interdependence in a cooperative setting is more likely to increase shared attitude and behavior than in a competitive setting, which supports the Social Interdependence Theory that positive interdependence increases prosocial behavior (14, 17). Sharing behavior is more likely to occur in a cooperative setting than a competitive one. One possible explanation is that playing games cooperatively in a group (positive independence) increases empathy (44). In particular, players who play cooperative games are affected by the expectation of others to reciprocate prosocial behaviors, which confirms the expectation of in-group members to reciprocate prosocial behaviors (45). However, playing competitive games (negative independence) may make individuals less sympathetic to their competitors, and they are less likely to take friendly actions toward them (46). Therefore, children exposed to games in a cooperative setting demonstrate higher levels of sharing attitude and behavior than those exposed to games in a competitive setting.

The second research hypothesis was not supported. Although we found a striking finding that Setting X Gender interactions were significant, the competitive setting increased girls’ sharing attitudes and behaviors, whereas the boys’ was relatively unaffected. We initially assumed that boys would exhibit higher levels of sharing attitude and behaviors than girls in a cooperative game setting, but we did not find gender differences in sharing attitudes and behaviors in a cooperative game setting. However, we only observed that girls exhibited significantly higher levels of sharing attitudes and behaviors than boys in a competitive game setting instead of a cooperative setting. This finding replicates previous research showing that girls show more prosocial tendencies than boys (29, 47). One possible explanation is that girls are more likely than boys to exhibit altruistic behavior in competitive games compared to cooperative games (30, 48). It is important to note that due to gender role categorization, girls in Chinese culture are often taught to be more concerned about and compliant with prosocial and antisocial behavior in the home and school settings than boys (49). Another assumption is that girls are more prosocial than boys at an early age. For example, girls are more prosocial than boys in early childcare centers (35). Although the competitive setting increased girls’ sharing attitudes and sharing behaviors compared to boys, the gender effects on sharing behavior was not significant in the cooperative setting. This finding also replicates previous studies that found weak sex differences in cooperation (17, 19, 50).

It is worth noting that we have not examined the age effects on sharing attitude and sharing behavior. Although previous studies have positively linked children’s prosocial development to age, suggesting that children become more generous as they get older (22, 30, 51), there are critics who argue that there are insignificant age differences in children’s sharing behavior (21). In our view, 4–5 year olds may be a relatively homogeneous group, and thus exhibit similar qualities in their sharing behavior. In addition, we found a significant positive correlation between sharing attitudes and sharing behaviors, supporting previous research that children exhibit prosocial behaviors after intentionally sharing with peers (52, 53). The possible explanation is that empathy and justice sensitivity affect altruistic sharing behavior (54). According to the theory of planned behavior, a more positive attitude will increase more positive behavior (55).

To our understanding, this experimental study may be the first to compare the effects of competitive and cooperative settings in the same game on sharing behavior among Chinese children. We believe this topic is interesting, and the findings are informative about the differential effects of competitive versus cooperative settings on sharing attitudes and behaviors. The novelty of this paper lies in the focus on culture and gender, and the research highlights of Eastern cultural effects are also interesting. The study has several notable strong points. First, the study was conducted under the Chinese context, which confirmed the fact that group-based competition and cooperation may be a ubiquitous social setting in kindergarten. Because both China and the West emphasize cooperation and competition. Second, the experimental methods and randomization of participants allowed us to draw causal conclusions about the positive effect of cooperative setting on children’s sharing behavior. Third, the manipulation of competitive vs. Cooperative game settings provides new insights into the cultivation of children’s sharing attitude and sharing behavior. In particular, the game settings and shared materials are close to the real lives of the children. Thus, this study may have good generalizability and ecological validity. Finally, the randomly selected non-Western sample supports the application of Social Interdependence Theory to young children in Western countries. Importantly, the main hypothesis that a cooperative game setting increases prosocial behavior in Social Interdependence Theory has been supported. Since the study sample in this study is in China, the findings of settings or cultural factors may provide useful information for this field. In this sense, our experimental work may advance science, practice, and policy related to the field of child psychology and behavioral development.

However, several limitations should be noted. First, there are typical problems of the sample. Only 120 children were recruited from two kindergartens, and the geographical area was closed, making it difficult to generalize to a wider group of children (e.g., urban vs. rural). Future studies may consider a broader group of to draw robust inferences. Second, although there is a positive correlation between sharing attitude and sharing behavior, we did not test the mediating effect of sharing attitude on sharing behavior, because sharing attitude is a dichotomous variable. Future research may test whether sharing attitudes mediate sharing behavior in children. Third, the reasonableness of game material selection is not enough. Although the game was screened by scoring, it is questionable whether “Pony Crossing” is a good operationalization of cooperation and competition. In particular, it is very likely that children may focus on speed rather than competition in the competitive mode, while children may focus more on rule avoidance than on true cooperation in the cooperative mode. Fourth, the hypothesis proposed (i.e., cooperative play increases sharing behavior) is an analogy based on adult research, but there may be different levels of social-cognitive development in unchildren and we failed to explore possible boundary conditions (e.g., children’s social-cognitive abilities, play experiences, etc.). we should consider this difference in the future. Finally, sharing behavior was measured in a simplistic manner, the measures should be improved. In this study, sharing was measured by the number of stickers, ignoring the motivation for sharing (e.g., voluntary vs. driven by social expectations) and the object of sharing (e.g., unfamiliar vs. familiar partners). In addition, participants performed a task with another child (i.e., a partner), and the participants were then asked whether they would share the reward with their partner. So this measure is more about perceived fairness than it is about prosocial behavior per se. Most importantly, the choice of sticker sharing as the basis for children’s willingness to share lacked rigor because, although children like stickers, they are a low-value, common item that is readily available in kindergartens or in everyday life (especially in China, where they are often distributed as rewards), and a child’s willingness to share does not necessarily mean that he or she would be willing to share something he or she considers to be of greater importance. Children’s willingness to share stickers may not be motivated by true prosocial motivation, but rather by the low personal value of the sticker to them and the low “cost” of sharing. This low-cost behavior is not representative of children’s willingness to share high-value items such as beloved toys or rare candies. Therefore, we need to provide them high-value items to ensure their true willingness to share them with others so as to improve scientific validity. Additionally, we did not discuss how competition and cooperation can be utilized together to better promote children’s development. So the results of the study may have limited value for application in practice.

This study has several implications. First, given that playing a game in the cooperative setting led to more sharing behavior than in the competitive setting, we should create more cooperative settings for children to increase their generous attitude and sharing behavior. For example, practitioners may offer more cooperative game lessons (e.g., skipping rope), whereas practitioners may forgo competitive game lessons or fully adversarial games. Parents can take their children to participate in parent-child activities, such as “You Draw, I Guess” and “Two People, Three Feet”, and so on. Children can cooperate with each other in the activity to form a positive interaction. For a family with two children, parents can ask their children to do some household tasks together, such as cleaning toys and sweeping the floor. Second, given that girls report more sharing than boys, especially in the competitive setting, practitioners should focus on cultivating boys’ sharing behavior. For example, we can create cooperative setting especially for boys to increase their sharing behavior. Finally, in view of the positive correlation between children’s sharing attitude and sharing behavior, practitioners can develop many training programs to increase sharing attitude, so as to further enhance children’s sharing behavior.

## Conclusion

To summarize, we attempt to conduct an experimental study to test the causal link between exposure to competitive versus cooperative game play and sharing behavior in Chinese children. In conclusion, our research expands previous literature on the effects of prosocial games on prosocial behavior by examining the effects of both competitive and cooperative settings of the same game on children’s sharing attitudes and sharing behaviors. Findings indicate that a cooperative game setting produces higher levels of sharing attitude and sharing behavior than a competitive game setting. Moreover, the most striking finding was Setting X Gender interactions. Basically, boys’ sharing attitudes and behaviors were unaffected by the Setting manipulation, whereas girls’ were affected. Similarly, the competitive setting increased girls’ sharing behavior, whereas the boys’ behavior was relatively unaffected. In particular, girls reported higher levels of sharing attitudes and behaviors than boys in a competitive setting. Thus, this study provides new evidence for boys as a target group that focuses on enhancing sharing behaviors in the competitive game setting. We also believe that these findings provide robust evidence of a prosocial game content effect and support for the Social Interdependence Theory.

## Data Availability

The raw data supporting the conclusions of this article will be made available by the authors, without undue reservation.
