# Supplementary figures and images for: Differential effects of exposure to cooperative versus competitive games on sharing behavior in young children
Source: Front Psychiatry. 2025 Jul 3;16:1545932. doi: 10.3389/fpsyt.2025.1545932 (PMC12268353; doi:10.3389/fpsyt.2025.1545932)

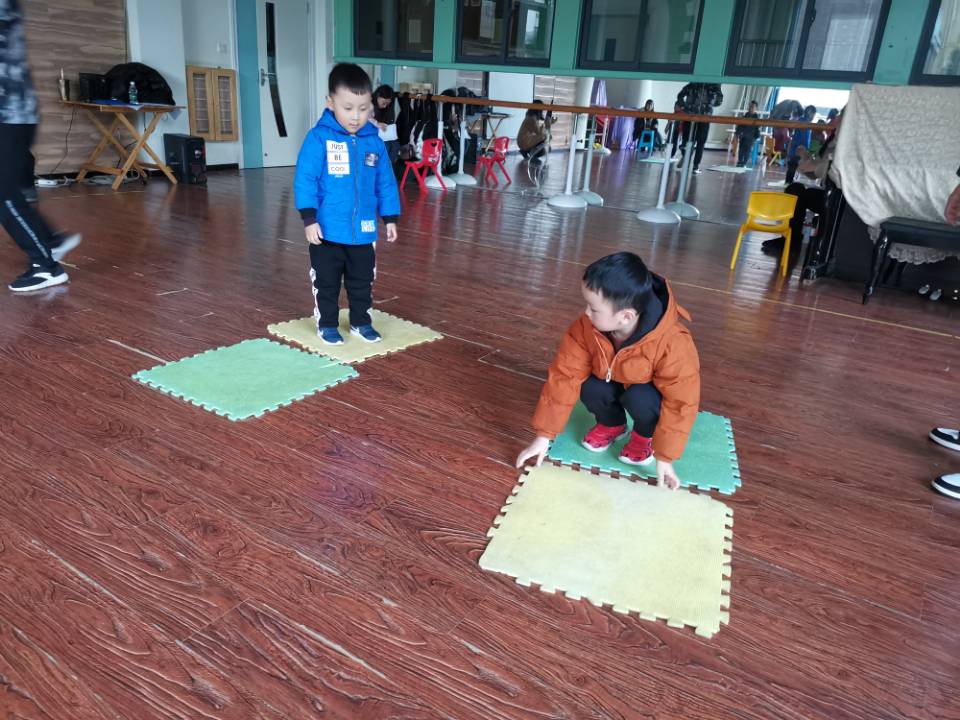

Supplement: Supplementary file 1 [file Image1.jpeg]

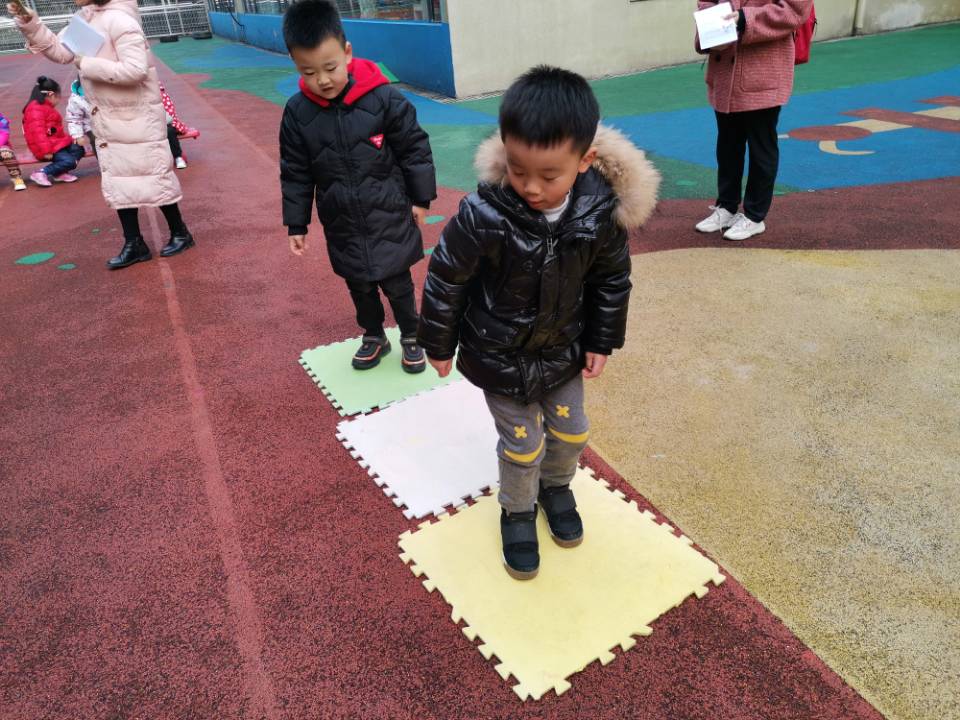

Supplement: Supplementary file 2 [file Image2.jpeg]
